# Nuclear survivin expression is a positive prognostic factor in taxane-platinum-treated ovarian cancer patients

**DOI:** 10.1186/1757-2215-4-20

**Published:** 2011-11-10

**Authors:** Anna Felisiak-Golabek, Alina Rembiszewska, Iwona K Rzepecka, Lukasz Szafron, Radoslaw Madry, Magdalena Murawska, Tomasz Napiorkowski, Piotr Sobiczewski, Beata Osuch, Jolanta Kupryjanczyk

**Affiliations:** 1 Department of Molecular Pathology, The Maria Sklodowska-Curie Memorial Cancer Centre and Institute of Oncology, Warsaw, Poland; 2 Chair of Gynaecologic Oncology, Medical University, Poznan, Poland; 3 Department of Biostatistics, The Maria Sklodowska-Curie Memorial Cancer Centre and Institute of Oncology, Warsaw, Poland; 4 Department of Anaesthesiology and Intensive Care, The Maria Sklodowska-Curie Memorial Cancer Centre and Institute of Oncology, Warsaw, Poland; 5 Department of Gynaecologic Oncology, The Maria Sklodowska-Curie Memorial Cancer Centre and Institute of Oncology, Warsaw, Poland; 6 Chair and Department of Obstetrics, Gynaecology and Oncology, IInd Faculty of Medicine, Warsaw Medical University and Brodnowski Hospital, Warsaw, Poland

**Keywords:** ovarian cancer, survivin, taxol, TP53

## Abstract

**Background:**

Survivin is an inhibitor of apoptosis and a regulator of mitotic progression. TP53 protein is a negative transcriptional regulator of survivin. The aim of our study was to evaluate the clinical significance of survivin expression in advanced stages ovarian cancer with respect to the TP53 status.

**Methods:**

Survivin and TP53 expression was evaluated immunohistochemically in 435 archival samples of ovarian carcinomas (244 patients were treated with platinum/cyclophosphamide-PC/PAC; 191-with taxane-platinum (TP) agents). Univariate and multivariate statistical analyses were performed in patients groups divided according to the administered chemotherapeutic regimen, and in subgroups with and without TP53 accumulation (TP53+ and TP53-, respectively).

**Results:**

Nuclear and cytoplasmic survivin expression was observed in 92% and 74% of the carcinomas, respectively. In patients treated with TP, high nuclear survivin expression decreased the risk of disease recurrence and death, and increased the probability of high platinum sensitivity (p < 0.01), but only in the TP53(+) group, and not in the TP53(-) group.

**Conclusions:**

It appears that TP53 status determines the clinical importance of nuclear survivin expression in taxane-platinum treated ovarian cancer patients.

## Background

Recently molecular anticancer therapies have undergone rapid development. Survivin, the smallest member of the family of the protein inhibitors of apoptosis (IAP) [1], is considered to be a potential target for molecular therapy [2]. Target survivin arose from data obtained from cancer cell lines, showing that survivin inhibition contributes to increased tumour response to various anticancer agents [3]. The results of clinical analyses are less consistent, as high survivin expression had been associated with both favourable and unfavourable prognosis [4].

Ovarian cancer is the most lethal gynaecological malignancy. In the last decade, taxanes combined with cisplatin or its analogues (TP therapy) have been considered standard first-line treatment for ovarian cancer [5,6]. Although the introduction of taxanes has significantly improved treatment results, still 20% to 30% of the patients fail to achieve complete remission [6-8].

Taxanes interact with β-tubulin and increase its polymerisation and stabilisation. In the presence of paclitaxel, cells form dysfunctional mitotic spindles and eventually die by apoptosis or necrosis (depending on drug concentration) [9,10]. The mechanism of action of taxanes is linked to survivin, which is a member of the chromosomal passenger complex (CPC) [11,12]. The CPC complex controls many aspects of mitosis, including regulation of the mitotic spindle checkpoint and mitotic progression [13]. It has been recently shown that, on treatment with taxol, survivin is involved in the spindle checkpoint activation and mitotic arrest [14,15]. Survivin, expressed during foetal development [16], and undetectable in most adult tissues [17] has been found in many types of human cancers, including ovarian cancer. The clinical role of survivin in ovarian cancer patients is not clear [18-20].

TP53 dysfunction enhances ovarian cancer response to taxane-platinum treatment [8,21,22]. In addition, the results recently obtained by our group suggest that the TP53 status, as determined by TP53 accumulation, may influence the clinical importance of other molecular factors [23-25]. This may also be the case with survivin, which is down-regulated by the wild-type TP53 [26,27]. Thus, there may be a synergistic clinical effect of TP53 dysfunction and high survivin expression in taxane/platinum-treated ovarian cancer patients.

We studied large groups of ovarian cancer patients in order to evaluate the clinical importance of survivin expression with respect to the TP53 status, and to the treatment regimen applied.

## Materials and methods

### Patients and tumours

The study was performed on 435 archival samples of ovarian carcinomas. Medical records were critically reviewed by at least two clinicians. The patients were treated with standard PC (cisplatin-cyclophosphamide or carboplatin-cyclophosphamide) or PAC chemotherapy (PC and doxorubicin) (244 patients), or with taxane-platinum chemotherapy (TP: paclitaxel or docetaxel with cisplatin or carboplatin) (191 patients). The material was carefully selected out of a total of 899 cases submitted to meet the following criteria: no chemotherapy before staging laparotomy, adequate staging procedure, International Federation of Gynaecologists and Obstetricians (FIGO) stage IIB to IV disease [28], tumour tissue from the first laparotomy available, moderate (G2) or poor tumour differentiation (G3 and G4), availability of clinical data, incl. residual tumour size and follow-up.

All tumours were uniformly reviewed histopathologically, classified according to the criteria of the World Health Organisation [29] and graded in a four-grade scale, according to the criteria given by Broders [30]. Clinicopathological characteristics have been presented in Table 1.

**Table 1:** Patients characteristics.

	ALL PATIENTS	TP-TREATED GROUP	PC/PAC-TREATED GROUP
	N = 435	N = 191	N = 244
**Age (years)**			
Range	20-78	20-78	24-77
Mean	54.3	54.9	53.9

**FIGO stage**			
IIB, IIC	27 (6%)	10 (5%)	17 (7%)
IIIA, IIIB	82 (19%)	26 (14%)	56 (23%)
IIIC	277 (64%)	136 (71%)	141 (58%)
IV	49 (11%)	19 (10%)	30 (12%)

**Residual tumour size**			
0	87 (20%)	35 (18%)	52 (21%)
≤ 2 cm	141 (32%)	77 (40%)	64 (26%)
> 2 cm	207 (48%)	79 (42%)	128 (53%)

**Histological type**			
serous	334 (77%)	142 (74%)	192 (79%)
endometrioid	22 (5%)	8 (4%)	14 (6%)
clear cell	15 (3%)	4 (2%)	11 (4%)
undifferentiated	33 (8%)	20 (11%)	13 (5%)
other types	31 (7%)	17 (9%)	14 (6%)

**Histological grade**			
G 2	54 (13%)	24 (12%)	30 (12%)
G 3	263 (60%)	110 (58%)	153 (63%)
G 4	118 (27%)	57 (30%)	61 (25%)

**Response to chemotherapy**			
complete remission	257 (59%)	124 (65%)	133 (55%)
partial remission/no change	112 (26%)	62 (32%)	50 (20%)
progression	66 (15%)	5 (3%)	61 (25%)

**Platinum sensitive**	129 (30%)	65 (34%)	64 (26%)
**Highly platinum sensitive**	83 (19%)	40 (21%)	43 (18%)
**Platinum resistant**	223 (51%)	86 (45%)	137 (56%)

**Number of patients at risk (OS)**			
1 year	389 (89%)	180 (94%)	209 (86%)
2 years	276 (63%)	141 (74%)	135 (57%)
3 years	172 (39%)	83 (43%)	89 (36%)
4 years	116 (27%)	53 (28%)	63 (26%)
5 years	76 (17%)	31 (16%)	45 (18%)

**Number of patients at risk (DFS)**			
1 years	143 (56%)	70 (56%)	73 (55%)
2 years	83 (32%)	40 (32%)	43 (32%)
3 years	61 (24%)	27 (22%)	34 (26%)
4 years	43 (17%)	17 (14%)	26 (19%)
5 years	28 (11%)	10 (8%)	18 (13%)

**Follow-up time**			
Range (month)	4.4-198.3	4.8-100.6	4.4-198.3
mean	30	32.2	27.5

**Outcome**			
NED	45 (10%)	27 (14%)	18 (7%)
AWD	45 (10%)	35 (18%)	10 (4%)
DOD	335 (77%)	124 (65%)	211 (87%)
DOC	10 (3%)	5 (3%)	5 (2%)

PC- cyclophosphamide and cisplatin, PAC-PC plus doxorubicin, TP-taxane-platinum therapy; NED-no evidence of disease, AWD-alive with disease, DOD-died of disease, DOC-died of other causes; OS-overall survival, DFS-disease free survival

For the PC/PAC-treated group, the follow-up time ranged from 4.4 to 198.3 months (median: 27.5); the respective values for the TP-treated group were: 4.8 to 100.6 months (median: 32.2). Short follow-up time resulted from early patients death. All surviving patients had at least a 6-month follow-up. Response to chemotherapy was evaluated retrospectively according to the World Health Organisation response evaluation criteria [31]. The evaluation was based on data from medical records describing patient's clinical condition and CA125 levels in 3-4 week intervals. Complete remission (CR) was defined as disappearance of all clinical and biochemical symptoms of ovarian cancer evaluated after completion of first-line chemotherapy and confirmed at four weeks. Within the CR group, we identified a platinum-sensitive group (PS, disease-free survival longer than six months) and a highly platinum-sensitive group (HPS, disease-free survival longer than 24 months). Other tumours were described as platinum resistant [32].

The study was approved by the bioethics committee of the Institute of Oncology (ref.no. 39/2007).

### Immunohistochemical analysis

Immunohistochemical stainings were performed on paraffin-embedded material after heat-induced epitope retrieval (HIER). We used a rabbit polyclonal anti-survivin antibody (1/1000, Novus Biologicals, Littleton, USA). TP53 protein was detected with the use of PAb1801 monoclonal antibody (1/3000, Sigma-Genosys, Cambridge, UK), as described previously [23]. The antigens were retrieved by heating the sections in 0.01 M citrate buffer (pH 6.0): 6 × 5 min. for survivin and 2 × 5 min. for TP53, at 700 W in a microwave oven. Non-specific tissue and endogenous peroxidase reactivities were blocked with 10% BSA and 3% H_2_O_2_, respectively. The sections were incubated with primary antibodies overnight, at 4°C. Biotinylated secondary goat anti-rabbit IgG (for survivin) (1/1500) and anti-mouse IgG (for TP53) (1/1500), peroxidase-conjugated streptavidin (1/500) (all from Immunotech, Marseille, France), and DAB were used as a detection system. As a positive control for TP53 accumulation, we used a tumour with a defined *TP53 *gene missense mutation [33]. Normal rabbit IgG or normal mouse IgG of the same subclasses and at the concentrations of the relevant primary antibodies served as negative controls.

### The evaluation of immunohistochemical stainings

Survivin expression was scored independently for nuclear and cytoplasmic staining. Light microscopic evaluation at 400× magnification was used to count at least 200 tumour cells within the areas of the strongest staining. Each nucleus in a given field was categorised according to the staining intensity (0 or weak to strong: 1 to 3), and counted. The nuclear survivin expression was further described as an ID score. It was calculated according to the following formula: ID = [(N0 × 0) + (N1 × 1) + (N2 × 2) + (N3 × 3)]/100, where N0, N1, N2 and N3 stands for the percentage of cells in each intensity category (0, 1, 2 or 3) [34]. A cytoplasmic staining was scored 0 (absent) or 1 to 3 (weak to strong). For the cytoplasmic expression, tumours with scores 0 and 1 were described as low expression, and those scoring 2 or 3, as high expression. Two independent assessors (A.R. and A.F-G.) concurred in 80% of the cases, and reached consensus in the remaining cases. TP53 protein accumulation was assessed as present (more than 10% positive cells) or absent, as previously described [8].

### Statistical analysis

The associations between survivin expression and clinicopathological variables were assessed using the chi-square test. Probability of survival and disease-free survival (DFS) were estimated using the Kaplan-Meier method and a log-rank test for censored survival data. Overall and disease-free survival time analyses were performed using multivariate Cox's proportional hazard models. Tumour response to chemotherapy (probability of CR, probability of PS or HPS) was evaluated using a multivariate logistic regression model.

Statistical analyses included the following independent variables: age of patients (median: 53 years), the FIGO stage, histopathological type and grade, residual tumour size and TP53 accumulation status. The survivin ID score was analysed as a continuous variable, and alternatively, as a categorical variable (cut-off point at median-1.5). Important factors were selected using a backward selection technique, where factors not significant at 0.1 were removed stepwise from the model.

The analyses were performed in the two groups of patients treated with different chemotherapeutic regimens, and additionally in the TP53(-) and TP53(+) subgroups. All tests were two-sided. P < 0.05 was considered significant. All calculations were performed using STATA 5 software.

## Results

### Cellular distribution of survivin expression

**Figure 1:**
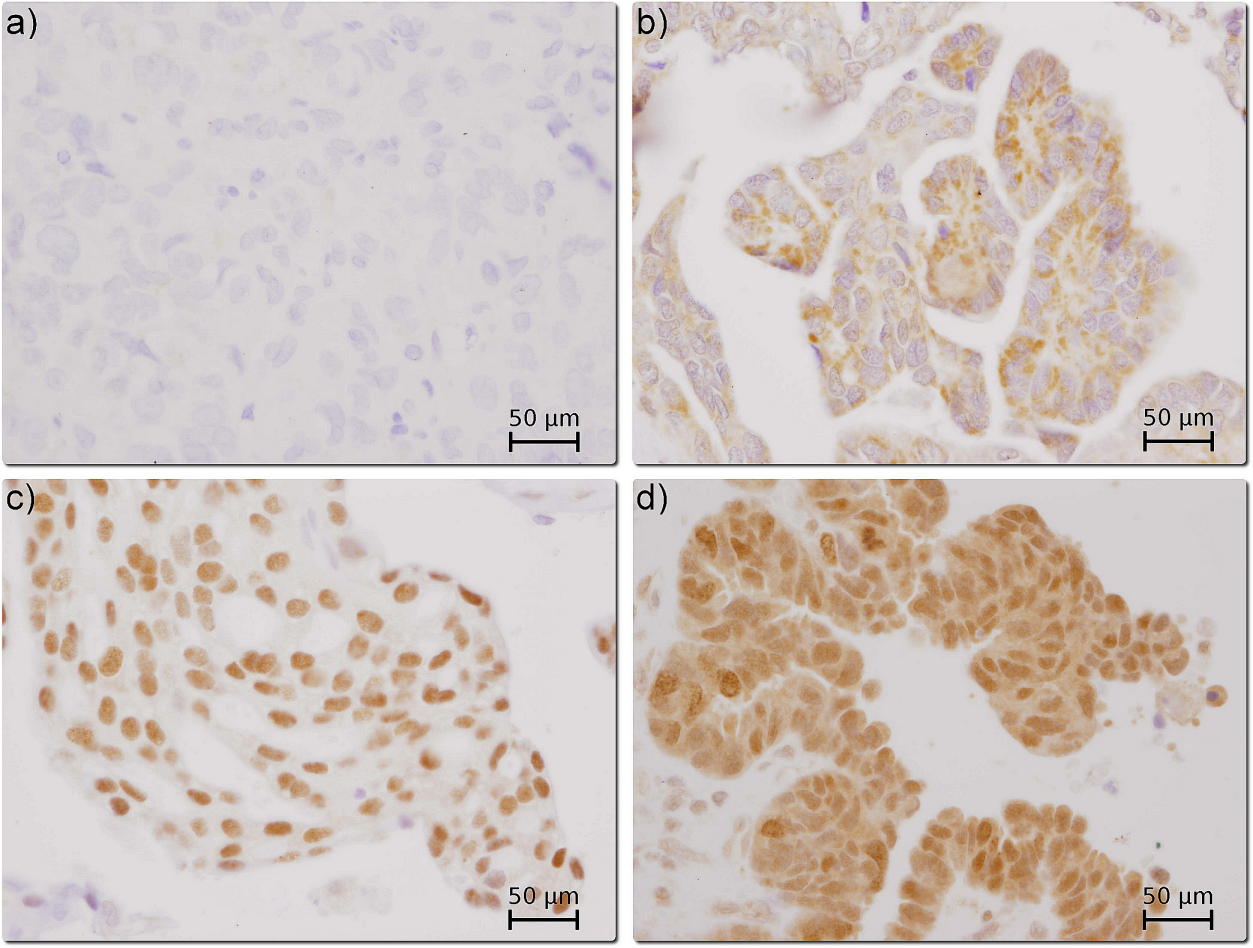
**Various patterns of survivin expression in four different ovarian carcinomas (400×, hematoxylin counterstain): a) negative survivin expression (clear cell carcinoma; FIGO IIIC), b) survivin expression absent in the nucleus but present in the cytoplasm (serous carcinoma; FIGO IIIB), c) survivin expression present in the nucleus only (serous carcinoma; FIGO IV), d) survivin expression present in the nucleus and cytoplasm (serous carcinoma; FIGO IV)**.

Survivin expression was observed both in the nuclei and the cytoplasm of ovarian cancer cells, predominantly in the former (Figure 1). Nuclear and cytoplasmic survivin expression of any intensity was observed in 92% and 74% of the tumours, respectively. Distribution of the nuclear and cytoplasmic survivin expression in all patients and in the subgroups treated with either chemotherapeutic regimen has been presented in Table 2.

**Table 2:** TP53 and survivin expression in ovarian carcinomas.

	ALL PATIENTS	TP-TREATED GROUP	PC/PAC-TREATED GROUP
	N = 435	N = 191	N = 244
**TP53-positive carcinomas**	255 (59%)	110 (58%)	145 (59%)
**TP53-negative carcinomas**	180 (41%)	81 (42%)	99 (41%)

**Cytoplasmic survivin expression:**			
Low (0 + 1 scores)	363 (83%)	149 (78%)	214 (82%)
High (2 + 3 scores)	72 (17%)	42 (22%)	30 (18%)

**Nuclear survivin expression:**			
ID score < 1.5	268 (62%)	97 (51%)	171 (70%)
ID score ≥ 1.5	167 (38%)	94 (49%)	73 (30%)

There were no associations between the cytoplasmic and nuclear survivin expression, nor between survivin expression and the clinicopathological variables studied nor TP53 accumulation.

### Survivin expression in the taxane/platinum-treated group

We analysed both cytoplasmic and nuclear survivin expression; only the nuclear expression related to the clinical endpoints. This was observed in the TP53(+) subgroup and, to a lesser degree, in the group comprising all patients, but not in the TP53(-) subgroup.

#### Analysis of survivin expression as a continuous variable

In the univariate analysis, high nuclear survivin expression positively influenced disease-free survival (HR 0.48, 95% CI 0.30-0.76, p = 0.002 for the TP53(+) group; HR 0.66, 95% CI 0.48-0.91, p = 0.013 for the entire group), overall survival (HR 0.63, 95% CI 0.43-0.91, p = 0.016 for the TP53(+) group only) and high platinum sensitivity (OR 4.25, 95% CI 1.57-11.51, p = 0.004 for the TP53(+) group; OR 2.40, 95% CI 1.27-4.53, p = 0.007 for the entire group). This was confirmed by the multivariate analyses (Table 3), in which the associations between survivin expression and the clinical endpoints were stronger and more significant in the TP53(+) group than in the entire group. In the TP53(+) group, high nuclear survivin expression apparently correlated with a lesser risk of recurrence (HR 0.44, p = 0.000) and death (HR 0.64, p = 0.010), and enhanced the odds of high platinum sensitivity (OR 5.04, p = 0.010) (Table 3).

**Table 3:** Associations of nuclear survivin expression (continuous variable) with clinical endpoints in the taxane-platinum-treated group* (multivariate Cox's proportional hazard and logistic regression models).

	All patients		TP53 (+) group	
	N = 199		N = 110	
	OR/HR [95%CI]	p-value	OR/HR [95%CI]	p-value
**High platinum-sensitivity^1^**				
Survivin expression	2.09 [1.04,4.17]	0.036	5.04 [1.47,17.18]	0.010
Residual tumour size				
0	1.0		1.0	
≤ 2 cm	0.17 [0.05,0.54]	0.003	-	
> 2 cm	0.09 [0.03,0.31]	0.000	0.21 [0.06,0.73]	0.014
Histological Grade				
Grade 2	-		1.0	
Grade 3	-		0.06 [0.00,0.74]	0.028
Grade 4	-		-	

**Disease-free survival**				
Survivin expression	0.67 [0.48,0.91]	0.013	0.44 [0.27,0.69]	0.000
Residual tumour size				
0	1.0		1.0	
≤ 2 cm	1.66 [0.99,2.78]	0.052	2.33 [1.13,5.26]	0.023
> 2 cm	1.88 [1.09,2.78]	0.022	3.09 [1.40,6.83]	0.005

**Overall survival**				
Survivin expression	-		0.64 [0.45,0.89]	0.010
Age (year)				
< 53	-		1.0	
≥ 53	-		1.68 [1.08,2.62]	0.020
FIGO stage				
II B, IIC	-		-	
III A, IIIB	-		-	
III C	-		1.0	
IV	-		2.76 [1.44,5.27]	0.002
Residual tumour size				
0	1.0		1.0	
≤ 2 cm	1.23 [1.22,4.07]	0.009	2.23 [1.06,4.67]	0.033
> 2 cm	3.41 [1.88,6.18]	0.000	2.99 [1.45,6.17]	0.003
Histological Grade				
Grade 2	1.0		1.0	
Grade 3	2.92 [1.45,5.86]	0.003	3.53 [1.45,8.61]	0.005
Grade 4	2.98 [1.44,6.15]	0.003	2.60 [1.01,6.69]	0.047

^1^HPS means the complete remission with DFS longer than 24 months.

* There were no associations with survivin ID score in the TP53(-) group.

In the TP53(+) group, the clinical importance of the survivin expression appeared stronger and more statistically significant than that of some clinicopathological factors (Table 3).

The residual tumour size was the clinicopathological factor most constantly associated with the clinical endpoints (Table 3). Complete remission and platinum sensitivity did not associate with nuclear survivin expression.

#### Analysis of survivin expression as a categorical variable

**Figure 2:**
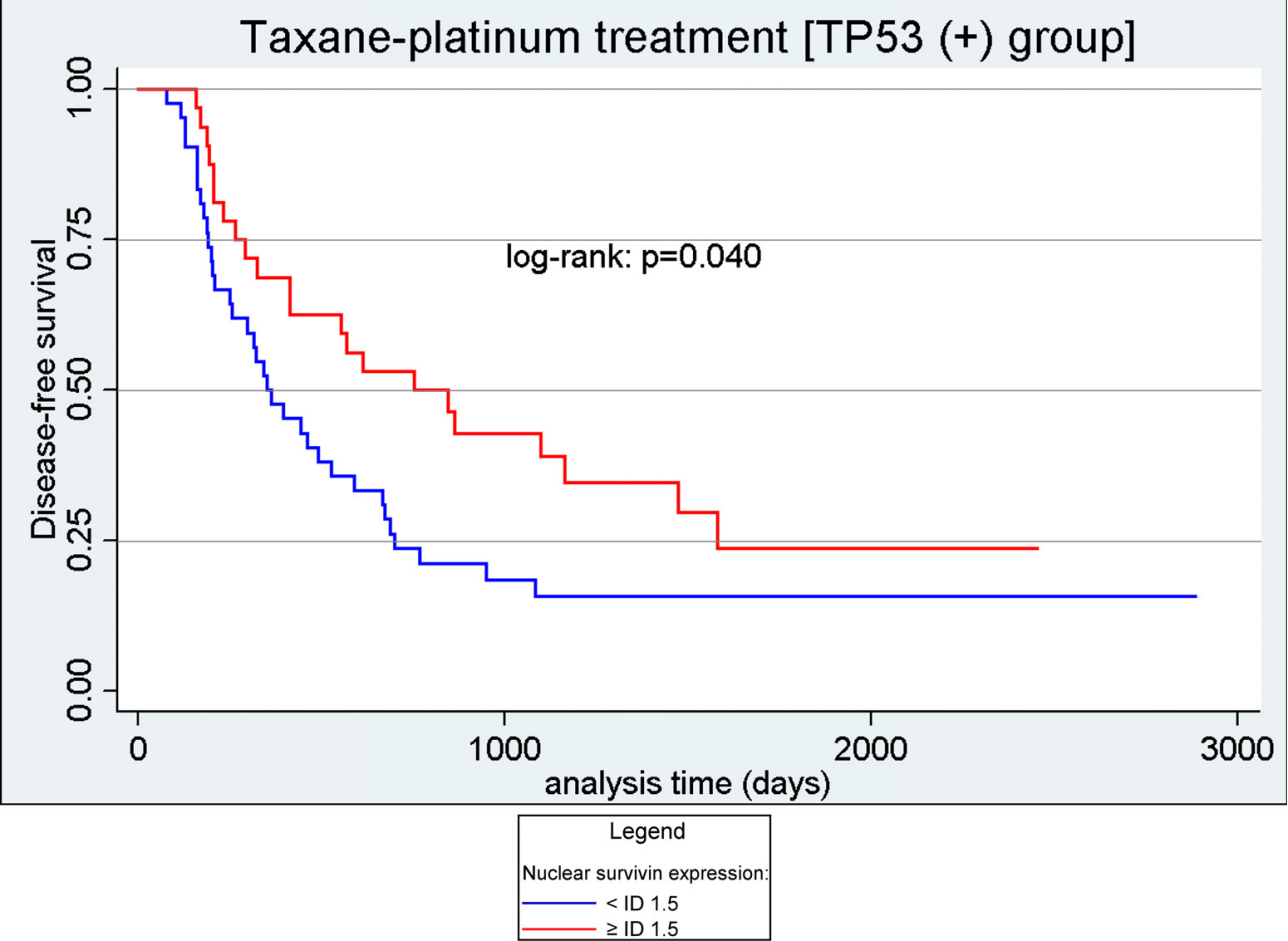
**Prognostic significance of nuclear survivin expression (Kaplan-Meier curve)**. Patients with high nuclear survivin expression (ID ≥ 1.5) had a significantly better disease-free survival than patients with low nuclear survivin expression (ID < 1.5).

In both univariate and multivariate analyses of the median survivin score (ID ≥ 1.5 vs < 1.5), the only association of survivin expression we observed was that with disease-free survival (univariate analysis: HR 0.57, 95% CI 0.33-0.98, p = 0.043 for the TP53(+) group [Figure 2]; multivariate analysis: HR 0.44, 95% CI 0.25-0.79, p = 0.005 for the TP53(+) group; HR 0.63, 95% CI 0.42-0.95, p = 0.029 for the entire group).

### Survivin expression in the platinum/cyclophosphamide-treated group

Neither nuclear nor cytoplasmic survivin expression has been found to correlate with clinical endpoints or clinicopathological factors in the PC/PAC patient group.

## Discussion

Survivin is regarded as a potential target of molecular therapy due to its strong antiapoptotic activity. Nevertheless, the results of numerous studies on the clinical importance of survivin in cancer patients are inconsistent. We present the first multivariate analysis which shows the positive prognostic significance of survivin expression in ovarian cancer patients. This was clearly demonstrated in patients treated with taxane-platinum agents, but not in those treated with platinum-cyclophosphamide regimens. In addition, the highest clinical significance of survivin was observed in patients with TP53 dysfunctional tumours.

A number of authors have reported, that the expression of survivin in cancer cell nuclei was associated with poor survival [35-37], while only a few studies have reported a reverse correlation [38-41]. Two studies on breast and lung cancer, have also shown the influence of nuclear survivin expression on DFS, similar to that observed in our analysis [39,41]. In case of ovarian cancer, the issue of survivin expression and tumour response to chemotherapy has been addressed by two groups [18,42]. One has not observed any correlation between nuclear or cytoplasmic survivin expression and the response to platinum/cyclophosphamide, but contrary to our results, they have not found any correlation with the response to TP therapy, either [18]. The other group has reported significantly higher rates of complete remission after taxol-based therapy in patients with low survivin-expressing tumours. However the latter group studied cytoplasmic survivin expression only [42].

Survivin may play a double role depending on its cellular localisation. In the cytoplasm, it exerts an antiapoptotic function by caspase inhibition. There is evidence to prove that nuclear survivin is a cell-proliferation promoting factor [43]. Studies conducted on different cell lines have shown, that survivin inhibition causes defects in cell division and suppresses proliferation. Some clinicopathological studies have identified positive correlations between nuclear survivin expression and various parameters of growth fraction (MIB-1, PCNA and mitotic indices) in hepatocellular carcinoma [reviewed in 4]. These findings point to survivin expression as an unfavourable prognostic factor. However, the majority of research groups evaluating the clinical importance of survivin expression have failed to consider the specific anti-tumour therapy applied. It should be stressed that in this study the positive prognostic significance of survivin expression was found only in patients treated with taxane-platinum agents. This relationship may be explained by a functional link between survivin and taxanes during mitosis [14,15].

Many studies regarding cell lines have revealed the influence of survivin expression on cancer sensitivity to taxanes. The studies that describe and/or explain the role of survivin in the mitotic checkpoint regulation are of particular interest. The data obtained from HeLa cells has shown that survivin is required for the maintenance of the spindle assembly checkpoint arrest in the presence of taxol, and this mechanism has been shown to be essential for taxol sensitivity [14,44]. In the presence of taxol survivin-depleted cells were unable to maintain the BubR1 protein at the kinetochores (BubR1 delays the transition to anaphase until all chromosomes are properly aligned). Exogenous expression of the wild-type survivin was able to restore the mitotic arrest-response of taxol-resistant cells [15]. Nevertheless, some of the studies analysing the biological effect of survivin suppression, or its overexpression in cell lines, have shown that survivin inhibited taxol-induced apoptosis [45,46]. Some authors have observed, that survivin overexpression (apparently the cytoplasmic survivin phosphorylated at Thr34) significantly decreased the sensitivity of human ovarian carcinoma cell lines to taxanes [42].

In our study patients with high survivin expression were at a lower risk of recurrence and death, but only in the TP53-positive group. As we have previously shown, the TP53 status may determine the clinical significance of the expression of other proteins, particularly of those regulated by, or interfering with TP53 in the control of tumour cell proliferation or apoptosis [23-25,47,48].

On the other hand, TP53 dysfunction positively influences cancer sensitivity to taxane-platinum therapy [8,22,49-51]. An increase in G2/M arrest or a loss of the TP53-dependent post-mitotic spindle checkpoint in the TP53 dysfunctional cells have been proposed as possible explanations of this phenomenon [52,53]. Thus, in view of the literature reports, the effects of TP53-dysfunction and high survivin expression may possibly show synergism, enhancing the response of cancer cells to taxol. The positive prognostic importance of survivin appears as a paradox. However, this may result from the survivin-dependent control of the mitotic response to taxol, rather than from antiapoptotic and proliferation-stimulating survivin activity.

TP53 may play a part in negative survivin regulation. Studies on cancer cell lines have shown that the wild-type TP53 repressed survivin at both mRNA and protein levels, by binding to its promoter [26,27]. Several authors have reported an association between dysfunctional TP53 status and high survivin expression. This has been shown in ovarian (high nuclear survivin in the TP53 mutant tumours), pancreatic, breast and gastric carcinomas [54-57]. We have failed to observe this correlation in our large group of 435 tumours; however, we evaluated TP53 accumulation only and it occurs with a frequency approximately 30% lower than TP53 mutations, thus the rate of TP53 dysfunctional tumours in our group may be much higher [33].

## Conclusions

In conclusion, our results show that the nuclear survivin expression status, in combination with the TP53 status, may be of prognostic value in ovarian cancer patients treated with taxane-platinum agents. The present study confirms our previous observations that analyses of carcinomas with and without TP53 accumulation, may play a pivotal role in the identification of cancer biomarkers.

## List of Abbreviations used

DFS: disease-free survival; HPS: high platinum sensitivity; ID: ID score; OS: overall survival; PC/PAC: platinum/cyclophosphamide chemotherapy; PS: platinum sensitivity; TP: taxane-platinum chemotherapy

## Competing interests

The authors declare that they have no competing interests.

## Authors' contributions

AFG designed and coordinated the study, participated in immunohistochemical analyses, drafted the manuscript. AR carried out immunohistochemical analyses. IKRz and LSz helped to draft the manuscript. MM carried out statistical analyses. TN, PS and BO, as well as the members of the POCSG collected and characterized the clinical material. JK participated in the design and coordination of the study, drafted and critically reviewed the manuscript. All authors have read and approved the final manuscript.
